# Allelic diversity of S-RNase alleles in diploid potato species

**DOI:** 10.1007/s00122-016-2754-7

**Published:** 2016-08-06

**Authors:** Daniel K. Dzidzienyo, Glenn J. Bryan, Gail Wilde, Timothy P. Robbins

**Affiliations:** 1 Plant and Crop Sciences Division, School of Biosciences, University of Nottingham, Sutton Bonington Campus, Loughborough, LE12 5RD UK; 2 Cell and Molecular Sciences, The James Hutton Institute, Invergowrie, Dundee, DD2 5DA Scotland, UK; 3 Biotechnology Centre, College of Basic and Applied Sciences, University of Ghana, P.O. Box LG 68, Legon-Accra, Ghana

## Abstract

**Key message:**

**The S-ribonuclease sequences of 16 S-alleles derived from diploid types of Solanum are presented. A phylogenetic analysis and partial phenotypic analysis support the conclusion that these are functional S-alleles.**

**Abstract:**

S-Ribonucleases (S-RNases) control the pistil specificity of the self-incompatibility (SI) response in the genus *Solanum* and several other members of the Solanaceae. The nucleotide sequences of S-RNases corresponding to a large number of S-alleles or S-haplotypes have been characterised. However, surprisingly, few S-RNase sequences are available for potato species. The identification of new S-alleles in diploid potato species is desirable as these stocks are important sources of traits such as biotic and abiotic resistance. S-RNase sequences are reported here from three distinct diploid types of potato: cultivated *Solanum tuberosum* Group Phureja, *S. tuberosum* Group Stenotomum, and the wild species *Solanum okadae.* Partial S-RNase sequences were obtained from pistil RNA by RT-PCR or 3′RACE (Rapid Amplification of cDNA Ends) using a degenerate primer. Full-length sequences were obtained for two alleles by 5′RACE. Database searches with these sequences identified 16 S-RNases in total, all of which are novel. The sequence analysis revealed all the expected features of functional S-RNases. Phylogenetic analysis with selected published S-RNase and S-like-RNase sequences from the Solanaceae revealed extensive trans-generic evolution of the S-RNases and a clear distinction from S-like-RNases. Pollination tests were used to confirm the self-incompatibility status and cross-compatibility relationships of the *S. okadae* accessions. All the *S. okadae* accessions were found to be self-incompatible as expected with crosses amongst them exhibiting both cross-compatibility and semi-compatibility consistent with the S-genotypes determined from the S-RNase sequence data. The progeny analysis of four semi-compatible crosses examined by allele-specific PCR provided further confirmation that these are functional S-RNases.

**Electronic supplementary material:**

The online version of this article (doi:10.1007/s00122-016-2754-7) contains supplementary material, which is available to authorized users.

## Introduction

The majority of flowering plants are hermaphrodite, and their reproductive organs are located in close proximity, a feature which might have imposed self-pollination and, subsequently, self-fertilisation on angiosperms. However, due to the deleterious effect of self-fertilisation and inbreeding, flowering plants have evolved several strategies to avoid this, the most widespread of which is self-incompatibility (de Nettancourt 1997, 2001). Self-incompatibility is a prezygotic barrier that enables the pistil, to distinguish self-pollen from non-self-pollen, leading to the arrest of self-pollen and thus blocking self-fertilisation (Kao and McCubbin 1996; Takayama and Isogai 2005). Depending on the genetic control of self-incompatibility in the pollen, plants are classified as having either a sporophytic or gametophytic mechanism (de Nettancourt 1977; Hiscock and McInnis 2003). The evolution of mechanisms to prevent inbreeding in flowering plants is partly responsible for their evolutionary success, thereby making them one of the most successful terrestrial groups of plants (Silva and Goring 2001).

Gametophytic self-incompatibility (GSI) represents the most prevalent form of self-incompatibility found in more than 60 flowering plant families. The Solanaceae, Rosaceae, Plantaginaceae, Leguminoceae, Onagraceae, Papaveraceae and Poaceae are amongst the plant families exhibiting this form of self-incompatibility (de Nettancourt 1977). The extensive study of GSI at the molecular level has revealed that it operates by two different mechanisms to achieve self-pollen recognition and rejection. One of these is the stylar ribonuclease (S-RNase) mechanism which has been initially identified and characterised in members of the Solanaceae, and later in the Rosaceae, Plantaginaceae and most recently in the Rubiaceae (Kao and McCubbin 1996; Nowak et al. 2011; Asquini et al. 2011). A distinct mechanism involving a pollen receptor is found in the Papaveraceae, in particular, *Papaver rhoeas* (Franklin-Tong and Franklin 2003; Wheeler et al. 2009). The pistil specificity of plant families exhibiting the S-RNase-based GSI system is controlled by polymorphic glycoproteins which are ribonucleases (S-RNases) and that have confirmed ribonuclease activity (Bredemeijer and Blass 1981; Anderson et al. 1986; McClure et al. 1989). Transgenic experiments in both petunia and tobacco have established that the S-RNase is the sole determinant of pistil specificity (Lee et al. 1994; Murfett et al. 1994).

Most of the diploid tuber-bearing *Solanum* species have a gametophytic system of self-incompatibility which is controlled by a single multi-allelic *S*-locus (Pushkarnath 1942; Pandey 1962; Cipar et al. 1964). Although phylogenetic studies of S-RNase diversity have been reported for several solanaceous species (Richman et al. 1996; Igic and Kohn 2001), this is yet to be conducted comprehensively in potato (*Solanum* subsection *Petota*) partly due to the relative paucity of S-RNase sequences available (see Table 4). Our aim in the present study was to identify and characterise additional S-RNase sequences in both cultivated and wild diploid potatoes based on breeding objectives as well as for the potential development of specialised genetic resources, such as populations of recombinant inbred lines (RILs). We have characterised S-alleles both phenotypically, using pollination tests, and genotypically, using an RT-PCR approach with degenerate primers. These led to the identification of new S-alleles in the primitive cultivated *Solanum tuberosum* Groups Phureja and Stenotomum, and the wild diploid species *Solanum okadae*. The identification and characterisation of these additional S-RNases in potato give an indication of the diversity of S-alleles in these taxa, thereby contributing significantly to the existing knowledge of the diversity of S-RNases in the Solanaceae family generally, and the genus *Solanum* subsection *Petota*, in particular.

## Materials and methods

### Plant materials

We use the classification of Dodds (1962) for describing primitive diploid cultivated potato germplasm. This scheme places the landrace germplasm studied here into two groups within *S. tuberosum*, Group Phureja and Stenotomum *.* Genebank accessions and breeding clones of *Solanum tuberosum* Group Phureja, *Solanum tuberosum* Group Stenotomum, and *Solanum okadae* (Table 1) are maintained at The James Hutton Institute (JHI, Invergowrie, Scotland) as part of the Commonwealth Potato Collection (CPC), and also duplicate clones were maintained during the course of these studies at the University of Nottingham. The plants were grown under controlled glasshouse conditions of 16 h photoperiod and 25 °C day/18 °C night temperatures during winter and natural day lengths during summer with supplementary lighting where necessary.

**Table 1:** Diploid potato species and clone designations used in this study

Potato species	Plant ID
*S. okadae*	OKA 7129-1
	OKA 7129-3
	OKA 7129-5
	OKA 7129-7
	OKA 7129-9
*S. stenotomum*	STN 4679
	STN 4679-68
	STN 4679-72
	STN 4711-61
	STN 4741
	STN 4741-119
	STN 4741-135
	STN 4786-80
*S. phureja*	DB 226-70
	DB 337-37
	DB 536-102

### Controlled pollinations

Controlled self- and cross-pollinations were carried out to confirm the SI status of the potato stocks and also to determine their possible compatibility relationships. The anthers of potatoes are hollow tubes (anther cones) that open by small apical pores. To collect pollen from these tubular anthers, a buzzer was used to vibrate the anther cone and the pollen collected into a microcentrifuge tube. The pollen was then deposited onto the stigma using a paint brush after the stigma had reached maturity. Crosses were scored approximately 4 weeks after pollination as either: self-incompatible if there were no berries (or no seed set) formed or fully self-compatible if berries (or seed set) could be seen following pollination.

### DNA extraction

Genomic DNA was extracted for allele-specific PCR genotyping from progenies obtained from *S. okadae* crosses that segregated for different S-alleles. Two leaf discs weighing approximately 10–100 mg of young leaves were processed for DNA extraction using the DNeasy Plant Mini kit (Qiagen, UK).

### Allele-specific PCR genotyping

The genotyping primers were designed to allow allele-specific amplification from plants segregating for known S-RNases. Specific primers used were as follows: S_*o1*_-RNase (*So1*-*F* (5′ GGATAAGGAGGGATCACAGC 3′) and *So1*-*R* (5′ TGTTGGCTTTGTATTTTGTAGCA 3′), S_*o2*_-RNase (*So2*-*F* (5′ TGCGAGTCCGAAGACAAGTA 3′) and *So2*-*R* (5′ AAGGGAAAGAAAACGGAAGC 3′)), S_*o4*_-RNase (*So4*-*F* (5′ TCGATTGGAGTTCTGCACTG 3′) and *So4*-*R* (5′TTTCATCGCATGTGTTACCC3′)) and S_*o5*_-RNase (*So5*-*F* (5′ TGGTCGAAAGGAACAACCTT 3′) and *So5*-*R* (5′ TTCCAACCTGGTCATTCAAAG 3′)). The primers were designed to have optimal melting temperature of 60 °C. PCR reactions were performed in a 25-μl reaction volume comprising 1X PCR buffer, 3 mM MgCl_2_, 0.2 mM dNTPs (Bioline), 0.4 μM of forward and reverse primers each and 2 U of Taq DNA polymerase (Bioline, London, UK). PCR amplification was performed in a PTC-200 Thermal Cycler (MJ Research, Watertown MA, USA) under the following cycling conditions: an initial 3-min denaturation at 94 °C, followed by 35 cycles of 30 s at 94 °C, 30 s of annealing at a temperature depending on the Tm (melting temperature) of the primer, 1 min at 72 °C and a final extension of 7 min at 72 °C.

### RNA extraction

RNA was extracted from pistil tissues using the RNeasy Plant Mini kit (Qiagen). Approximately 10–100 mg of tissue, pre-chilled in liquid nitrogen (N_2_), was ground to a fine powder and processed for the RNA extraction.

### Reverse Transcription (RT)-PCR reaction: 3′RACE

Reverse Transcription (RT) reactions were carried out using an oligo-dT primer (NotI d(T)18) (5′-AACTGGAAGAATTCGCGGCCGCAGGAA(T)_18_-3′) consisting of a 27-bp anchor part with a *Not*I restriction site (included at the 5′ end) and 18 thymidine (T) nucleotides at the 3′end. First-strand cDNA synthesis was achieved using Omniscript^®^ Reverse Transcriptase (Qiagen). The reaction comprised 1X RT Buffer, 0.5 mM dNTPs, 1 µM NotI d(T)18 primers, 10 U of RNase inhibitor (RNaseOut Recombinant Ribonuclease Inhibitor, Invitrogen), 4 U of Omniscript Reverse Transcriptase and approximately 1–2 µg of total RNA template denatured at 65 °C for 5 min in RNase-free water. The final RT reaction mixture was incubated at 37 °C for 1 h for cDNA synthesis.

A degenerate primer, *SolC2*-*F1.3* (5′-TTTACNRTNCATGGNCTNTGGCC-3′) was designed based on the C2 conserved domain of Solanaceae S-RNases (Ioerger et al. 1991). This was used to amplify partial S-RNase sequences from pistil RNA using the RT-PCR-based 3′ RACE (Rapid Amplification of Complementary DNA Ends) technique. The degenerate primer (*SolC2*-*F1.3*) was used together with another primer (*Not*I-anchor primer) (5′-AACTGGAAGAATTCGCGG-3′) which has the same recognition sequence as the anchor part of the oligo-dT primer (*Not*I d(T)18) used in the first-strand cDNA synthesis. RT-PCR amplification was performed in a 25-µl total reaction mix comprising a 2.5-µl aliquot of RT reaction (cDNA reaction), 20 mM Tris–HCl (pH 8.3), 50 mM KCl, 1.5 mM MgCl_2_, 0.2 mM dNTPs (Bioline), 0.4 µM of *SolC2*-*F1.3* (forward), 0.2 µM *Not*I-anchor (reverse) primers and 2 U of *Taq* DNA polymerase (Bioline). Amplification was performed as described previously with an annealing temperature of 55 °C and a final extension of 5 min.

### Reverse transcription (RT)-PCR reaction: 5′RACE

Gene-specific primers were designed from the partial sequences isolated from the 3′RACE cloning for the identified S-RNases and used for full-length cDNA cloning using the 5′RACE-PCR technique. Three gene-specific primers (GSPs): *So2*-*GSP1* (5′-ATTATACCATGATTTCGGAGAGC-3′), *So2*-*GSP2* (5′-AGATCGATACTACACGTTCCATG-3′) and *So2*-*GSP3* (5′-CAGTGATACTCCAGTTGTTTGC-3′) were used for cloning full-length *S*_*o2*_-RNase. For the *S*_*s2*_-RNase, *Ss2*-*GSP1* (5′-AGAAGTAATACCATTCTTTCCGAG-3′), *Ss2*-*GSP2* (5′-AACACGTTCCATGCTTAATG-3′) and *Ss2*-*GSP3* (5′-CCAGATGTATTCCAGAGCTTC-3′) gene-specific primers were used. First-strand cDNA synthesis and 5′RACE-PCR technique was carried out using the 5′RACE System for Rapid Amplification of cDNA Ends, Version 2.0 (Invitrogen) following the manufacturers’ recommendations. PCR products were obtained using AAP (abridged anchor primer) or AUAP (abridged universal anchor primer) primers (supplied in the kit) and the gene-specific primers (GSPs) for each allele.

### Cloning into pCR^®^2.1 vector

RACE-PCR products were cloned using the TA cloning kit (Invitrogen). The concentration of the PCR product (less than 1 day old) needed to ligate with 50 ng (20 fmoles) of pCR^®^2.1 vector was determined, and a 10-μl ligation reaction was set up with 1 µl of 10× ligation buffer, 2 µl of 25 ng pCR^®^2.1 vector and 1 µl of 4.0 Weiss units T4 DNA ligase. The ligation mixture was incubated at 14 °C overnight, transformed into One Shot competent cells (TOP10F’ Invitrogen) and plated on LB plates containing 40 mg/ml of X-Gal, 100 mM of IPTG and either 50 µg/ml of kanamycin or 100 µg/ml of ampicillin.

### Sequencing of plasmid inserts

Colonies were screened using M13 universal primers, and plasmid DNA was extracted from putative positive clones using the QIAprep Spin Miniprep kit (Qiagen). The sequence of the inserted DNA was determined using forward and reverse M13 universal primers at either the Qiagen Genomic Services/Sequencing Services (Qiagen) or The James Hutton Institute sequencing facility (JHI, Invergowrie, Scotland).

### Analysis of S-RNase sequences

The alignment, comparison and phylogenetic analysis of the putative S-RNase sequences were performed using the sequence data analysis tools listed below. DNASTAR software (DNASTAR Inc., Madison, USA) was used to deduce the amino acid sequence of the cloned putative S-RNases. The deduced amino acid sequences were then subjected to the NCBI database BLAST (http://blast.ncbi.nlm.nih.gov) searches, and the accession numbers of the best hits were noted. The accession numbers were then entered into the EBI database (http://srs.ebi.ac.uk) for the retrieval of the amino acid sequence of four of these best hits where necessary. The cloned S-RNases were aligned together with one of the selected S-RNase sequences from the database to act as a reference gene. Alignments were performed by ClustalW method (Thompson et al. 1994) using BioEdit (http://www.mbio.ncsu.edu/BioEdit/bioedit) and also MegAlign™ as implemented in DNASTAR using the default settings and edited manually. The percentage similarities of the deduced amino acid sequence of the cloned S-RNases were also determined by calculating the sequence distances using the neighbour-joining method (Saitou and Nei 1987) on the basis of the alignment using ClustalW method as implemented in MegAlign™ (DNASTAR). Phylogenetic analysis of the cloned S-RNase sequences together with selected solanaceous S-RNases and S-like-RNases retrieved from the EBI database (http://srs.ebi.ac.uk) was performed using the neighbour-joining tree method as implemented in MEGA5 software (Tamura et al. 2011). The evolutionary distances used to infer the phylogenetic tree were calculated using a Poisson correction method (Zuckerkandl and Pauling 1965) as implemented in MEGA5. To show how well the topology of the tree was supported, bootstrap analysis (Felsenstein 1985) was performed using 1000 replicates. Three *Antirrhinum* S-RNase amino acid sequences (accession numbers X96464, X96465 and X96466) were also included in the phylogenetic tree as an ‘out-group’ to root the tree, and in other cases, T2-RNase of *Aspergillus oryzae* (accession number CAA43400.1) was used to root the phylogenetic tree. Positions which contain gaps and missing data were eliminated from the phylogenetic analysis. All S-RNase sequence data have been submitted to the NCBI database with accession numbers listed in Supplementary Table S4.

## Results

### Confirming the SI status and the compatibility relationships of potato germplasm

To confirm the SI status and the compatibility relationships among some of the potato germplasm used in this study, pollinations were performed following a diallel cross design. The results from clones within the *S. okadae* accession OKA7129 (Table 2) showed that all self-pollinations resulted in no berries set (self-incompatible). From the crosses, it was observed that two of the OKA7129 clones are likely to be harbouring the same pair of S-alleles based on incompatible cross-pollinations. Crosses between OKA1 and OKA3 consistently failed to produce any berries in either direction indicating that these clones share the same pair of S-alleles. Crosses involving the other parents were observed to set seed in either direction and could represent either a compatible or a semi-compatible cross (see supplementary list S1 for the full details of the crosses). The compatibility relationship of other potato plants used in this study (Group Stenotomum and Group Phureja) could not be checked using pollination tests due to the limited number of flowers available or lack of flowering time synchronisation.

**Table 2:** Pollination data for *S. okadae*

Female parent	Male parent				
	OKA1	OKA3	OKA5	OKA7	OKA9
OKA 1	*	*	106	125	125
OKA 3	*	*	50	71	82
OKA 5	19	60	*	37	87
OKA 7	52	50	106	*	90
OKA 9	113	115	100	79	*

The figures in the table are the average numbers of seed set per berry (seeds/berry) for the various crosses. The asterisks (*) represent the failed (incompatible) crosses. Accession names in Table 1 are abbreviated e.g. OKA1 = OKA7129-1

### RT-PCR cloning of pistil S-RNases

**Fig. 1:**
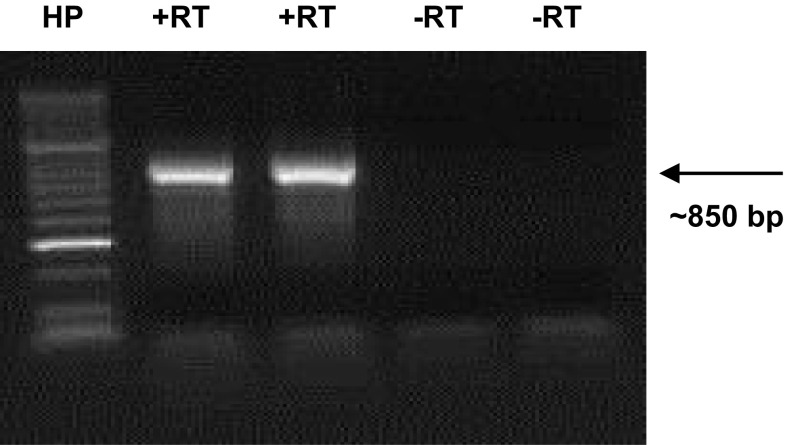
Representative example of RT-PCR of pistil S-RNases in *Solanum okadae*. RT-PCR of OKA 9 diploid potato pistils using a consensus S-RNase C2 domain primer. *Lane* *+RT* represents duplicate samples with RT (Reverse Transcriptase), *−RT* represents duplicate samples without RT. *Lane* *HP* represents Hyperladder II size marker (Bioline)

A degenerate primer was designed for the C2 conserved domain based on the alignment of solanaceous S-RNases and used in combination with an oligo-dT *Not*I-anchor primer. This enabled the amplification by RT-PCR of putative pistil S-RNases from *S. tuberosum* Group Stenotomum, *S. tuberosum* Group Phureja and *S. okadae* resulting in the expected amplicon size of ~ 850 bp for all three taxa (data not shown). An example of such an RT-PCR for *S. okadae* OKA9 is given in Fig. 1. The first-round RT-PCR gave a discrete size of PCR product for almost all accessions tested and, hence, were used directly for cloning without the need for a nested PCR step. To enable the selection of colonies having the expected insert size, colony PCR using M13 universal forward and reverse primers was performed. An example of the colony PCR results obtained with accession OKA9 is shown in Supplementary Fig. 1. Three such colonies were selected for each S-RNase cloning experiment, and plasmid DNA was purified for sequencing.

### Sequencing and alignment of the 3′RACE products

The sequencing results produced a total of 17 putative S-RNases from genotypes of the three taxa studied following database searches. These S-RNases were provisionally called S_*o1*_ to S_*o5*_ for those from *S. okadae*, S_*s1*_ to S_*s10*_ for those from Group Stenotomum and S_*p1*_ and S_*p2*_ for those from Group Phureja (Table 3). The S-RNase designation is based on the order in which they were identified in each of the species. Results from the NCBI database searches revealed that our cloned S-RNases shared sequence similarity with S-RNases from other solanaceous species (data not shown). When the S-RNases were initially cloned, only four S-RNases from *Solanum chacoense* could be found that were similar (S_*2*_, S_*3*_, S_*11*_ and S_*14*_). However, during the later stages of this research, additional sequences for potato species (*Solanum* subsection *Petota*) were submitted to NCBI and found to be similar to the cloned alleles. Database mining for S-RNases published in potato to date (December 2015) has revealed a total of 17 S-RNase sequences (Table 4). However, there were no exact matches of our cloned S-RNases to the previously published alleles indicating that they are all novel.

**Table 3:** Summary of the sixteen deduced S-genotypes of *Solanum* accessions used in the study

Species	Plant ID	Cloned S-allele				Deduced S-genotype
		Allele	Freq*	Allele	Freq*	
*Solanum okadae*	OKA 1	S_*o1*_	3	S_*o2*_	4	S_*o1*_S_*o2*_
*Solanum okadae*	OKA 3	S_*o1*_	2	S_*o2*_	3	S_*o1*_S_*o2*_
*Solanum okadae*	OKA 5	S_*o1*_	13	S_*o5*_	2	S_*o1*_S_*o5*_
*Solanum okadae*	OKA 7	S_*o3*_	10	–	–	S_*o3*_S_*o*_?
*Solanum okadae*	OKA 9	S_*o1*_	7	S_*o4*_	2	S_*o1*_S_*o4*_
*Solanum stenotomum*	STN 4679	S_*s1*_	3	S_*s9*_	2	S_*s1*_S_*s9*_
*Solanum stenotomum*	STN 4679-72	S_*s5*_	4	–	–	S_*s5*_S_*s*_?
*Solanum stenotomum*	STN 4711-61	S_*s2*_	3	S_*s3*_	2	S_*s2*_S_*s3*_
*Solanum stenotomum*	STN 4741	S_*s4*_	8	S_*s10*_	1	S_*s4*_S_*s10*_
*Solanum stenotomum*	STN 4741-135	S_*s8*_	4	–	–	S_*s8*_S_*s*_?
*Solanum stenotomum*	STN 4786-80	S_*s6*_	3	S_*s7*_	4	S_*s6*_S_*s7*_
*Solanum phureja*	DB 226	S_*p1*_	4	–	–	S_*p1*_S_*p*_?
*Solanum phureja*	DB 337	S_*p2*_	4	–	–	S_*p2*_S_*p*_?
*Solanum phureja*	DB 536	S_*p2*_	4	–	–	S_*p2*_S_*p*_?

* Freq cloned frequency of each allele observed from the total number of colonies examined

**Table 4:** S-RNases for potato retrieved from NCBI database

Potato species	S-allele	Accession #	References
*S. chacoense*	S_*2*_	X56896.1	Xu et al. (1990)
*S. chacoense*	S_*3*_	X56897.1	Xu et al. (1990)
*S. chacoense*	S_*11*_	S69589.1/L36464.1	Saba-el-Leil et al. (1994)
*S. chacoense*	S_*12*_	AF176533.1/AF191732.1	Qi et al. (2001)
*S. chacoense*	S_*13*_	L36667.1	Despres et al. (1994)
*S. chacoense*	S_*14*_	AF232304	O’Brien et al. (2002)
*S. chacoense*	S_*16*_	DQ007316	Marcellan et al. (2006)
*S. stenotomum*	S_*3*_	HM446648	Kear (2010), unpublished
*S. stenotomum*	60_A	AEN02425.1	Kear and Malinski (2010), unpublished
*S. stenotomum*	60_B	AEN02426.1	Kear and Malinski (2010), unpublished
*S. stenotomum*	60_C	AEN02427.1	Kear and Malinski (2010), unpublished
*S. stenotomum*	60_D	AEN02428.1	Kear and Malinski (2010), unpublished
*S. stenotomum*	60_E	AEN02429.1	Kear and Malinski (2010), unpublished
*S. stenotomum*	47_A	AEN02423.1	Kear and Malinski (2010), unpublished
*S. stenotomum*	47_D	AEN02424.1	Kear and Malinski (2010), unpublished
*S. phureja*	S_*36*_	HM446649	Kear (2010), unpublished
*S. tuberosum*	S_*2*_	X62727	Kaufmann et al. (1991)

Some of the plants from which we have isolated S-RNases harbour the expected two S-alleles, whilst only one allele could be found in others, notably the Phureja group genotypes (Table 3). Attempts were made to identify the second allele of those in which only one could be found by further rounds of colony PCR which proved successful for some but not all genotypes studied. For instance, a second allele was identified for OKA9, but examination of a similar total number of clones examined for OKA7 was not successful in identifying a second allele. In an attempt to identify the second allele of these accessions, a negative screening approach was taken. Allele-specific primers were designed and used to screen all colonies revealed by M13 primers to have inserts with the expected sizes. Those that did not amplify with the allele-specific primers were thought to be candidates for the putative second allele of the accessions screened and were sent for sequencing. However, after screening large numbers of colonies (typically ~50–100), only a few of these putative second alleles could be identified. The previously cloned allele from which the primers were designed was present in almost all the colonies screened for the accessions with just one allele (data not shown), suggesting that the allele-specific primers did not amplify the original allele efficiently in every case.

From the sequencing results summarised in Table 3, it is apparent that some alleles occur in more than one accession. For example the S_*o1*_-RNase was found to be present in four of the *S. okadae* genotypes from accession OKA7129, i.e. OKA1, OKA3, OKA5 and OKA9. Similarly, the S_*o2*_-RNase was also found to be present in both OKA1 and OKA3. Interestingly, S_*s9*_ and S_*p1*_ were found to be present in both the Stenotomum accession STN 4679 and Phureja clone DB226(70), respectively. This allele was initially cloned from Stenotomum and named S_*s9*_ and was later cloned from Phureja and named S_*p1*_ (see Table 3). However, sequence comparison showed that, these two S-RNases shared exactly the same deduced amino acid sequence (data not shown) and, hence, were provisionally renamed S_*s9*_/S_*p1*_ (or S_*s9p1*_) to represent both alleles although they could theoretically represent two functionally distinct alleles. This observation is not unexpected as Stenotomum and Phureja are closely related evolutionarily and are considered members of the same *Solanum tuberosum* species group (Spooner et al. 2014). Allowing for this identity, the total number of novel S-RNase sequences identified in this study is 16 comprising 5 from *S. okadae* and 11 from *S. tuberosum* Groups Stenotomum and Phureja.

**Fig. 2:**
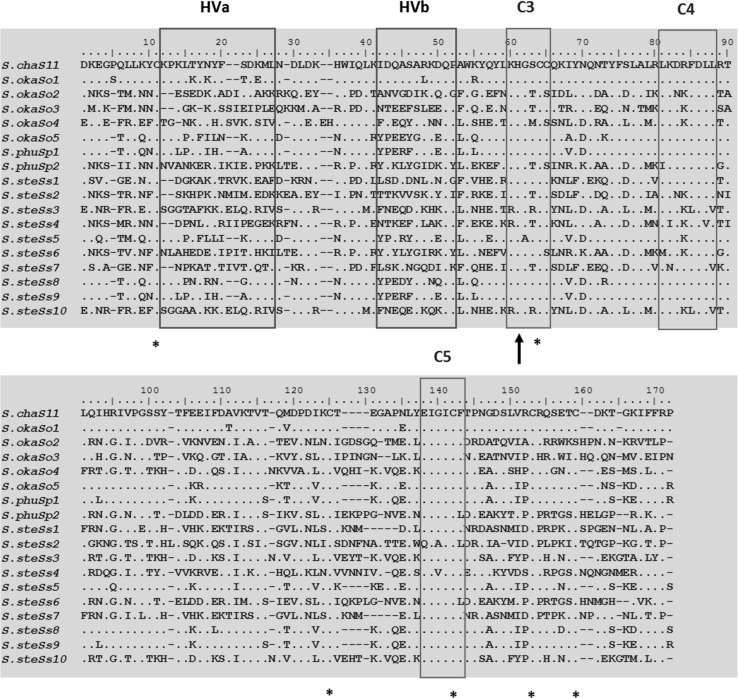
Alignment of the deduced amino acid sequence of the 16 novel S-RNases cloned from *S. okadae, S. phureja* and *S. stenotomum* with one published S-RNase, S11 from *Solanum chacoense* (Acc. No: S69589.1). The hypervariable regions (HVa and HVb) and conserved regions (C3–C5) are *boxed*. The *dots* in the alignment indicate identities among the 16 sequences with reference to the S11-RNase. *Gaps* in the alignment are indicated by (−). The conserved histidine residue in the C3 region involved in the ribonuclease activity of S-RNases is *marked with an arrow head*. Six conserved cysteine residues are marked with *asterisks* (*) under the alignment

The alignment of the deduced partial amino acid sequence of the 16 novel putative *Solanum* S-RNases is shown in Fig. 2. Three of the conserved domains (C3–C5) and the two hypervariable domains (HVa and HVb) are highlighted and are part of the primary structural features of solanaceous S-RNases as defined by Ioerger et al. (1991). One of the two catalytic histidine residues (His) known to be involved in the ribonuclease activity of S-RNases is indicated in the alignment of the C3 region. Six out of the eight conserved cysteine residues found in selected solanaceous S-RNases (Ioerger et al. 1991) are also indicated. These cysteine residues are important for determining the tertiary structure of S-RNases (Ishimizu et al. 1996). The remaining two were not shown because they are located in regions which are outside of this alignment (i.e. between C1 and C2). However, one of the cysteine residues shown in this alignment was not perfectly conserved. Thus, all sixteen cloned S-RNases contained all six cysteine residues expected with the exception of the *S*_*o2*_-RNase which lacks a residue at the 3′ end (i.e. the last conserved cysteine located just after the C5 region). This particular cysteine residue is also missing in four other solanaceous S-RNases published in public databases (accession numbers AAB26702.1, CAA53666.1 and BAC00933.1 and AAV69976.1).

The percentage amino acid sequence similarity among the 16 S-RNases ranged from 32.9 to 94.5 % (Table 5). This low level of sequence similarity is consistent with the high level of polymorphism known to exist between S-RNase alleles in the Solanaceae (Ioerger et al. 1990). From the table, it could be observed that amino acid similarity within a species could be as low as 32.9 % for the *S. okadae* S-RNases (S_*o1*_ vs S_*o2*_), 33.1 % for the *Stenotomum* S-RNases (S_*s2*_ vs S_*s3*_) and 44.2 % for the two Phureja S-RNases. The isoelectric point (p*I*) value was calculated for the fully and partially deduced amino acid sequences using DNASTAR software, and the values revealed that all the S-RNases are basic proteins and have a p*I* value in the range of 8.6–9.6 (data not shown).

**Table 5:** Percentage amino acid similarity of the sixteen cloned S-RNases

*S*-allele	*S*_*o1*_	*S*_*o2*_	*S*_*o3*_	*S*_*o4*_	*S*_*o5*_	*S*_*s1*_	*S*_*s2*_	*S*_*s3*_	*S*_*s4*_	*S*_*s5*_	*S*_*s6*_	*S*_*s7*_	*S*_*s8*_	*S*_*s9p1*_	*S*_*s10*_	*S*_*p2*_
*S*_*o1*_	–	32.9	42.2	52.3	73.9	43.7	33.5	50.6	41.9	72.0	38.3	41.1	75.8	76.4	51.3	39.6
*S*_*o2*_		–	47.5	38.0	34.8	44.6	48.4	34.4	45.3	37.4	44.1	42.0	35.5	35.5	34.4	45.3
*S*_*o3*_			–	42.3	44.8	44.2	37.7	39.2	43.8	44.8	42.1	43.6	44.2	44.2	39.9	44.7
*S*_*o4*_				–	53.5	40.3	38.0	70.8	40.5	54.8	44.6	38.3	56.8	56.1	72.7	44.6
*S*_*o5*_					–	39.7	33.5	50.6	39.4	84.7	43.5	40.4	86.0	86.6	50.0	44.2
*S*_*s1*_						–	42.9	35.9	39.5	39.1	37.2	74.5	39.7	38.4	36.5	37.2
*S*_*s2*_							–	33.1	43.5	34.8	36.2	45.5	33.5	33.5	35.0	38.8
*S*_*s3*_								–	40.6	53.8	38.4	34.0	53.2	52.6	94.5	40.9
*S*_*s4*_									–	40.6	43.8	40.1	41.9	41.3	42.5	48.8
*S*_*s5*_										–	43.5	37.7	82.8	84.1	53.2	42.9
*S*_*s6*_											–	37.8	41.6	42.2	40.3	80.4
*S*_*s7*_												–	39.7	38.4	36.5	39.1
*S*_*s8*_													–	87.9	53.8	42.9
*S*_*s9p1*_														–	53.8	44.2
*S*_*s10*_															–	42.1
*S*_*p2*_																–

Species of origin for S-alleles are abbreviated as follows: *S*_*o*_ = *S. okadae*, *S*_*s*_ = *S. stenotomum*, *S*_*p*_ = *S. phureja*

### 5′RACE of selected S-RNases

**Fig. 3:**
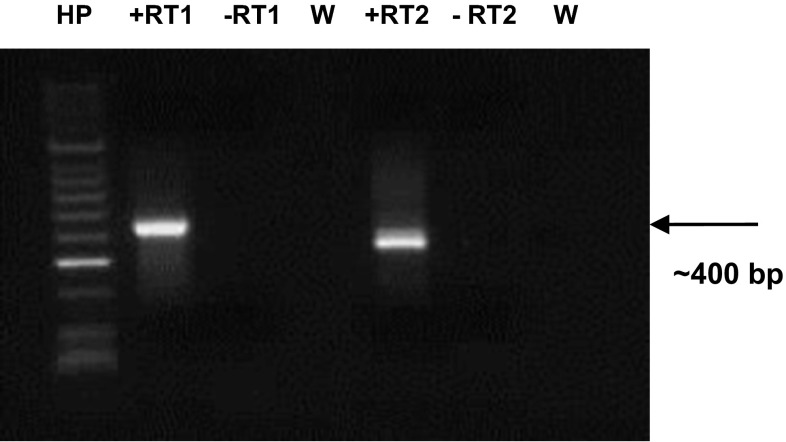
5′RACE-PCR of So2-RNase from OKA 1. *Lane HP* represents Hyperladder II (bioline), *+RT1* represents sample with RT (reverse transcriptase) from 1st round PCR, *−RT1* represents sample without RT from 1st round PCR, *+RT2* represents sample with RT from 2nd round (nested) PCR, sample *−RT2* represents sample without RT from 2nd round PCR, *W* represents water control

An attempt was made to clone the full-length sequence of two selected alleles, i.e. S_*o2*_-RNase from *S. okadae* and S_*s2*_-RNase from Stenotomum, using 5′ RACE. Three antisense gene-specific primers (GSPs) were designed from the partial sequence obtained through the 3′RACE cloning and used in 5′RACE to enable the full-length S-RNase gene sequence to be determined. The RT-PCR products gave the expected amplicon in both cases as indicated for the S_*o2*_-RNase in Fig. 3. Following transformation, three colonies having the expected insert size as revealed by colony PCR (Supplementary Fig. 2) were selected for plasmid DNA extraction and sequencing of inserts.

### Sequencing and alignment of the 5′RACE products

**Fig. 4:**
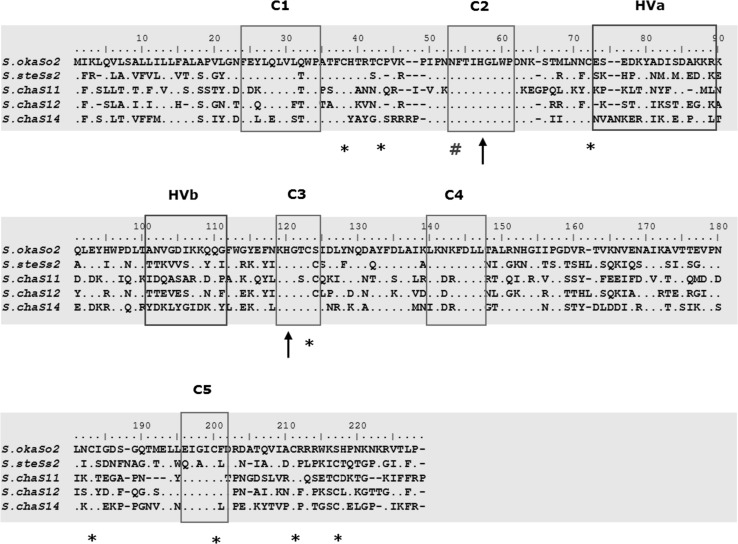
Alignment of the deduced amino acid sequence of the full gene sequence of So2-RNase (S. okaSo2) cloned from *S. okadae* and Ss2-RNase (S. steSs2) from *S. stenotomum* with three published full-length S-RNases from *S. chacoense*. The hypervariable regions (HVa and HVb) and conserved regions (C1–C5) are *boxed*. The *dots* in the alignment indicate similarities between the five sequences. The conserved histidine residues involved in the ribonuclease activity of S-RNases are *marked with arrow heads*. The eight conserved cysteine residues are marked with an *asterisk* (*) under the alignment. The only conserved potential N-glycosylation site found in solanaceous S-RNases is marked with a *hash symbol* (#) under the alignment

The 5′RACE cloning allowed the addition of the C1 and C2 domains to the 5′ region of the two selected S-RNases. The C1 and C2 domains were not part of the initial partial sequence alignment of the cloned S-RNases (Fig. 2). The amino acid sequence alignment of the two full-length cloned S-RNases (S_*o2*_ from *S. okadae* and S_*s2*_ from Group Stenotomum) with three of the published full-length *S. chacoense* S-RNase sequences from the database is shown in Fig. 4. The two hypervariable regions (HVa and HVb) and the five conserved domains (C1, C2, C3, C4 and C5) found in all solanaceous S-RNases are indicated. The two conserved histidine residues which are located on the C2 and C3 domains and known to be involved in the ribonuclease activity of S-RNases are also indicated with an arrowhead in the alignment. The eight conserved cysteine residues found in solanaceous S-RNases (Ioerger et al. 1991) are also indicated, with the exception of S_*o2*_-RNase which has only seven cysteine residues as explained previously. Also, the only conserved single potential N-glycosylation site found within C2 in most solanaceous S-RNases (Ioerger et al. 1991) is shown in the alignment.

### Phylogenetic analysis of S-RNases

**Fig. 5:**
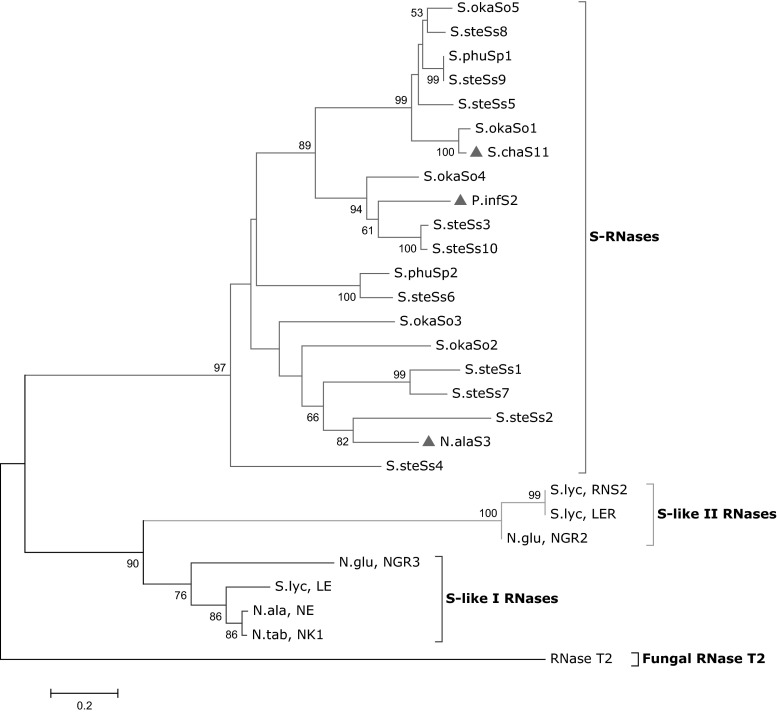
Phylogenetic tree of S-RNases and S-like RNases. A phylogenetic tree of the 16 cloned S-RNases and selected S-like RNases from Solanaceae. Three published S-RNases, one from each of *Petunia*, *Nicotiana* and *Solanum chaoense* (highlighted with a *triangle*) were included as reference sequences. Fungal RNase T2 of *Aspergillus oryzae* was included as an out-group to root the phylogenetic tree. *Numbers* are bootstrap values expressed as a percentage and only those exceeding 50 % are shown. Bootstrap values were based on 1000 replicates. The phylogenetic tree was drawn using MEGA5 software. N.ala = *Nicotiana alata*, N.glu = *Nicotiana glutinosa*, N.tab = *Nicotiana tabacum*, S.lyc = *Solanum lycopersicon*, S.oka = *Solanum okadae*, S.phu = *Solanum phureja*, S.ste = *Solanum stenotomum*

Phylogenetic trees were constructed using the neighbour-joining method as implemented in MEGA5 (Tamura et al. 2011). Bootstrap values of the tree were calculated based on 1000 replicates to allow an estimate of how well the topology at a branch on the tree is supported. Plant T2-type RNases have been classified into three categories: class I, II and III. All S-RNase genes identified to date belong to the class III type/group (Igic and Kohn 2001; Nowak et al. 2011), and type I and II groups are non-S-RNases and are generally referred to as S-like I and S-like II, respectively. In an attempt to confirm that our cloned S-RNases belong to the class III RNase group and are genuine S-RNases and not S-like-RNases (as suggested from the Blast search), a phylogenetic tree was constructed based on an alignment using our cloned S-RNases and selected class I and II RNase members from the Solanaceae (Fig. 5). The resulting phylogenetic tree showed that the putative S-RNases cluster together with typical class III S-RNases used for reference and are distinct from the clades of S-like-RNases. The fungal RNase (T2-RNase) which shares homology with S-RNases (and from which the name T2-type RNase was derived) was used as an out-group to root the phylogenetic tree (see supplementary list S2 for the accession numbers of the selected S-like-RNases used). Although there are rare examples of sequences that group with S-RNases in phylogenenetic analysis that have been shown to be non-polymorphic and unlinked to the S-locus (e.g. Lee et al. 1992), this is exceptional, and, generally, this association is considered as good evidence for the identification of genuine S-RNases (Igic and Kohn 2001).

**Fig. 6:**
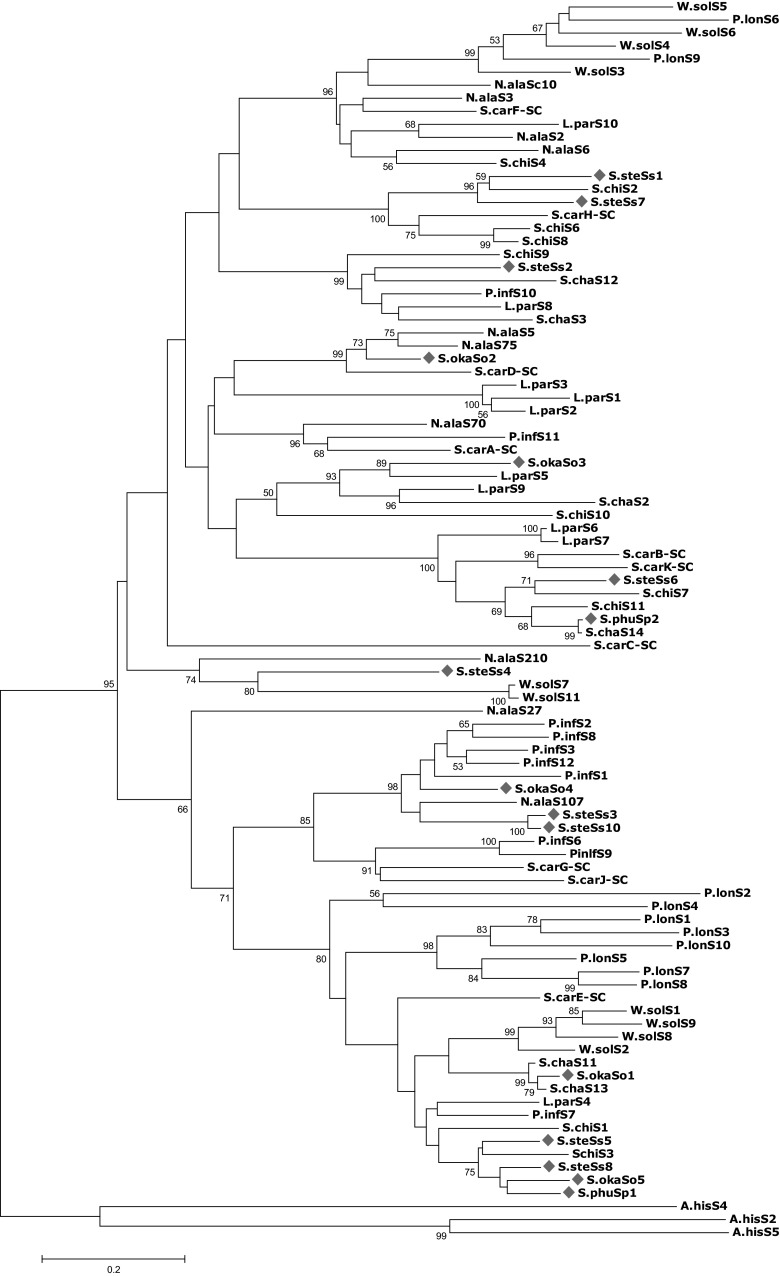
Phylogenetic tree of selected S-RNases from the Solanaceae and the S-RNases reported in this study. The 16 cloned S-RNases are indicated with red diamonds. Three *Antirrhinum* S-RNases are included as an out-group to root the phylogenetic tree. Other sequences are selected solanaceous S-RNases retrieved from databases. *Numbers* are bootstrap values expressed as a percentage and only those exceeding 50 % are shown. Bootstrap values were based on 1000 replicates. Phylogenetic tree was drawn using MEGA5 software. *Solanum* (potato) S-RNases are labelled in red, *Solanum* (tomato) S-RNases are in green, *Solanum carolinense* S-RNases are in black, *Petunia* S-RNases are in blue, *Lycium* in fuchsia, *Nicotiana* S-RNases in purple, *Witheringia* *solanacae* S-RNases are in aqua, *Physalis* S-RNases are in lime and *Antirrhinum* S-RNases are in maroon. A.his = *Antirrhinum hispanicum*, L.par = *Lycium parishii*, N.glu = *Nicotiana glutinosa*, P.inf = *Petunia inflata*, P.lon = *Physalis longifolia*, S.car = *Solanum carolinense*, S.chac = *Solanum chacoense*, S.chil = *Solanum chilense*, S.oka = *Solanum okadae*, S.ste = *Solanum stenotomum*, W.sol = *Witheringia solanacae*

An expanded phylogenetic analysis to cover a more comprehensive sample of S-RNases in the Solanaceae is shown in Fig. 6. This phylogenetic tree is based on an alignment involving the partially deduced amino acid sequence of the 16 cloned S-RNases and a sample of 70 selected solanaceous S-RNases retrieved from public databases (see supplementary list S3). This sample included 10 S-alleles from each of seven representative species from the genera: *Solanum, Nicotiana, Petunia, Physalis, Lycium* and *Witheringia*. Three *Antirrhinum* S-RNase sequences are also included as an ‘out-group’ to root the phylogenetic tree (Xue et al. 1996). The topology of the phylogenetic tree showed that the cloned potato S-RNases are dispersed across many solanaceous lineages. Also, interspecific similarities rather than intraspecific similarities are often observed among the S-RNases, i.e. the cloned S-RNases do not often cluster into species-specific clades but rather form clades with alleles from other members of the Solanaceae that are supported by high bootstrap values in most cases. For example, S.okaSo2 is part of a strongly supported clade with two alleles from *Nicotiana alata*, one from *Solanum carolinense*, and none from *Solanum okadae*. In some cases, the S-RNases can be observed to be clustered with S-RNases from exclusively other genera of the Solanaceae rather than forming genus-specific clades. For example, S.steSs4 is part of a clade with alleles of *Witheringia* and *Nicotiana* but no *Solanum* alleles identified to date. The clustering of the S-RNases of one species/genus with members of another species/genus rather than the same species/genus is a commonly observed feature of solanaceous S-RNases reflecting the ancient origin and diversification of the S-RNases in the common ancestors of the Solanaceae (Ioerger et al. 1990).

### Progeny analysis with allele-specific primers

**Fig. 7:**
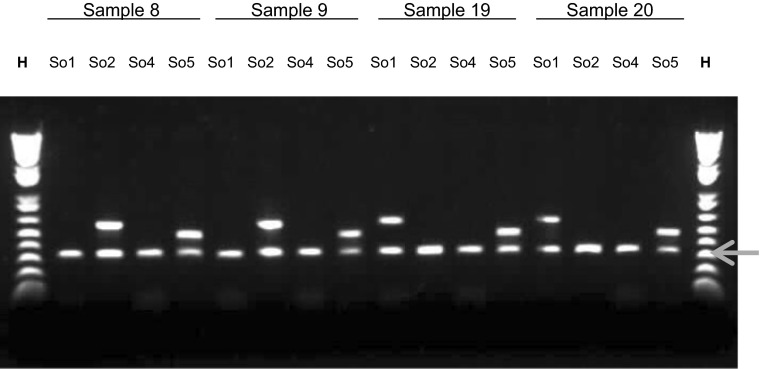
Progeny analysis of semi-compatible crosses with allele-specific primers. S-allele genotyping of a selection of four progeny (*samples 8, 9, 19* and *20*) from the cross OKA1 (*S*_*o1*_ *S*_*o2*_) × OKA5 (*S*_*o1*_ *S*_*o5*_). Each DNA sample was amplified with allele-specific primers for the *S*_*o1*_, *S*_*o2*_, *S*_*o4*_ and *S*_*o5*_ alleles, respectively, as indicated. *Samples 8* and *9* were positive for *S*_*o2*_ and *S*_*o5*_, whilst samples 19 and 20 were positive for *S*_*o1*_ and *S*_*o5*_. The *reference band* indicated with a *red arrow* at approximately 230 bp is the result of an internal control PCR. The full genotype analysis of this family based on the genotype of 30 individuals is shown in Table 6

To test S-allele function, a selection of semi-compatible crosses was performed in *Solanum okadae.* Progeny plants of four crosses between genotypes OKA1, OKA3, OKA5 and OKA9 were analysed for the presence of the expected S-RNase alleles. Allele-specific primers were designed for each of the four alleles represented in these genotypes (*S*_*o1*_, *S*_*o2*_, *S*_*o4*_ and *S*_*o5*_) and used in PCR amplifications from each of 28–30 progeny plants per cross. The allelic constitution of these plants is summarised in Table 6, and an example showing the PCR genotyping of S-RNases from four such individuals is shown in Fig. 7. In each case, the individual plant genotypes were consistent with a ‘semi-compatible’ reaction, whereby pollen containing an allele held in common with the pistillate parent did not fertilise the female parent. All progeny plants contained the ‘non-common’ S-allele from the pollen donor parent in a heterozygous state. For example, in the first cross in Table 6 (OKA1 × OKA5), the *S*_*5*_ allele of the pollen parent is inherited by all progeny. Conversely, the *S*_*1*_ allele of the pollen parent is efficiently rejected as no progeny of *S*_*1*_*S*_*1*_ or *S*_*1*_*S*_*2*_ genotype was identified. All 116 genotyped progenies are consistent with the transmission of the expected single staminate S-RNase allele. This provides good evidence that the cloned sequences are equivalent to functional S-alleles showing gametophytic control of the pollen phenotype such that matching alleles are efficiently rejected during pollination and do not appear in the zygotes formed.

**Table 6:** Analysis of progenies resulting from *S. okadae* crosses by PCR using allele-specific primers

Cross name	Number of progeny individuals evaluated	Parental genotypes (♀) × (♂)	Expected genotypes	Number of obtained genotypes
OKA 1 × OKA 5	30	(*S*_*1*_ *S*_*2*_) × (*S*_*1*_ *S*_*5*_)	*S*_*1*_ *S*_*5*_	13
			*S*_*2*_ *S*_*5*_	17
OKA 1 × OKA 9	29	(*S*_*1*_ *S*_*2*_) × (*S*_*1*_ *S*_*4*_)	*S*_*1*_ *S*_*4*_	18
			*S*_*2*_ *S*_*4*_	11
OKA 3 × OKA 5	29	(*S*_*1*_ *S*_*2*_) × (*S*_*1*_ *S*_*5*_)	*S*_*1*_ *S*_*5*_	16
			*S*_*2*_ *S*_*5*_	13
OKA 3 × OKA 9	28	(*S*_*1*_ *S*_*2*_) × (*S*_*1*_ *S*_*4*_)	*S*_*1*_ *S*_*4*_	14
			*S*_*2*_ *S*_*4*_	14

## Discussion

### SI status confirmation and the compatibility relationships of potato germplasm

The SI mechanism in angiosperms enables the female reproductive organ of a flower to recognise and distinguish between self-pollen and non-self-pollen and, hence, to allow only the non-self-pollen to effect fertilisation. In the GSI system, depending on the nature of the S-haplotypes expressed in both the pollen and the pistil, different compatibility relationships could be observed. Thus, a compatible or semi-compatible reaction will occur when at least one of the haplotypes expressed in the pollen and pistils do not match. If the two parents differ by just one haplotype, then a semi-compatible reaction will occur, and all the pollen tubes carrying the shared haplotype will be arrested, whilst those carrying the unique haplotype will be accepted. Alternatively, when both of the expressed S-haplotypes in the pollen and pistil are matched, it will result in an incompatible reaction leading to the arrest of all the growing pollen tubes.

The diallel cross design used here has established the compatibility relationships among the five *S. okadae* genotypes studied (Table 2). For instance, crosses between OKA1 and OKA3 did not yield any berries or seeds in either direction. This implies that both parents harbour the same pair of S-haplotypes, hence, the arrest of all growing pollen tubes results in an incompatible reaction. Crosses among all the other *S. okadae* genotypes resulted in the production of berries and seeds in either direction. From these crosses, two plausible compatibility relationships could be inferred. The first is a compatible reaction (cross) where the parents involved harbour different pairs of S-haplotypes and, therefore, should not lead to the arrest of the growing pollen tube. Alternatively, the parents could differ by at least one S-allele in a semi-compatible cross where one of the alleles go through to effect fertilisation, whilst the one shared by both parents will be arrested. It should be noted that all of the self-pollinated *S. okadae* plants did not yield any berries/seeds, thereby confirming that the accessions are, indeed, self-incompatible.

The S-genotypes inferred from the S-RNase sequence analysis in *S. okadae* (Table 3) are entirely consistent with the pollination data. OKA1 and OKA3 are shown to have the same genotype S_*o1*_S_*o2*_ consistent with the reciprocal cross-incompatibility observed between these two lines. The S-genotyping further suggests that the crosses between OKA1 and OKA3 and the other three lines studied should be either semi-compatible (OKA5 and OKA9) or possibly fully compatible (OKA7). The RT-PCR cloning could not identify a second S-RNase allele in OKA7, possibly due to a divergence from the consensus C2 sequence, in which case the crosses would be fully compatible. The seed set observed in all these crosses in Table 2 supports this interpretation, and it was not possible to distinguish semi-compatibility and full compatibility based on the amount of seed set. This suggests that pollen is available in excess such that all possible ovules are fertilised even in a semi-compatible pollination.

### Progeny analysis of semi-compatible pollinations

To further test the above interpretation, a series of four *Solanum okadae* semi-compatible progenies were genotyped at the S-RNase locus using allele-specific primers. The four allele-specific primers were designed to amplify all four possible S-alleles expected to be segregating in the progenies of the respective crosses. In all the crosses, a semi-compatible reaction was expected since a common S-allele, S_*o1*_-RNase, was present in all the parents involved in the crosses (Table 6). The S-genotyping results for a total of 116 progeny are consistent with the pollination data (Table 2) and the deduced S-RNase analysis sequence data (Table 3), such that the expected segregation of S-alleles was observed in the progenies of the respective crosses. The observations from the allele-specific PCR genotyping are consistent with the expectation that all the pollen tubes carrying the shared S-haplotype are arrested, whilst those carrying the unique S-haplotype are accepted. For example, in the first cross in Table 6 (*S*_*1*_*S*_*2*_ × *S*_*1*_*S*_*5*_), there were no progeny of *S*_*1*_*S*_*2*_ or *S*_*1*_*S*_*1*_ genotype identified, consistent with the rejection of the *S*_*1*_ haplotype in pollen. These crosses provide good evidence that the cloned S-RNase sequences in *S. okadae* represent functional S-alleles.

### Primary structural features of S-RNases in selected diploid potato plants

Relatively few S-RNase sequences are available for potato species (*Solanum* subsection *Petota*) compared to other members of the Solanaceae. As of December 2015, the NCBI database mining for S-RNases revealed only 17 S-RNases for potato (Table 4), most of which were cloned from either *S. chacoense* or *S. tuberosum* Group Stenotomum. There are some additional S-alleles from *S. tuberosum* mentioned in the literature (Kirch et al. 1989; Kaufmann et al. 1991), however, not all of them are in public databases. The use of the C2 domain degenerate primer and the 3′ RACE strategy described here has enabled the cloning of an additional 16 novel putative S-RNases from accessions of three diploid potato plants; *S. okadae*, *S. tuberosum* Group Stenotomum and *S. tuberosum* Group Phureja. Plants exhibiting the S-RNase-based GSI system are expected to be heterozygotes bearing two different S-alleles. However, the observation that only one allele could be cloned from some of the accessions could be explained by the differential amplification of the S-RNases by the degenerate primer. Alternatively, the unidentified S-RNases may have very low transcript levels relative to the other allele and, hence, could not be detected or amplified. Alleles at the S-locus can show significant variation in their transcript levels or their stability (Roldan et al. 2010). All the identified putative S-RNases are novel, and no cDNA sequences were available for them prior to this study. The 16 alleles reported here are a significant addition to the existing number of S-RNase sequences reported for tuber-bearing members of *Solanum* (subsection *Petota*), almost doubling the number available in public databases.

Analysis of the partially deduced amino acid sequence obtained here (Fig. 2) showed that all the cloned sequences have the primary structural features of solanaceous S-RNases as originally defined by Ioerger et al. (1991). Also, the cloned partial S-RNase sequences contain one of the active site histidine (His) residues located in the C3 region which are involved in the ribonuclease activity of the S-RNase gene. The other histidine which is located in the C2 region is not part of the partial sequences obtained for most of the S-RNases cloned here, because the degenerate primer used for the cloning is from the C2 region and, hence, was removed from all partial sequences due to nucleotide sequence ambiguity in that region. The cloned partial S-RNase sequences contain six out of the eight conserved cysteine residues found in functional S-RNases (with the exception of S_*o2*_-RNase) which can form potential disulphide bonds (Ioerger et al. 1991). The partial S_*o2*_-RNase sequence was observed to contain only five of the expected six cysteine residues. This observation was not unprecedented since this particular cysteine residue is absent from some published functional solanaceous S-RNases, notably the four best matches for the S_*o2*_-RNase from the NCBI database searches (i.e. three S-RNases from *S. peruvianum* with database accession numbers AAB26702.1, CAA53666.1 and BAC00933.1 and one from *N. glauca* with database accession number AAV69976.1). The formation of disulphide bridges by the cysteine residues is considered vital for forming and stabilising the tertiary structure of the proteins (Ishimizu et al. 1996; Ida et al. 2001) although it appears that at least one disulphide bridge is dispensible. All of the cloned putative S-RNase proteins (full and partial sequences) were predicted to have strong basic isoelectric point values (8.6–9.6), which is consistent with observations made with functional S-RNases involved in the self-incompatibility reaction, i.e. functional S-RNases are basic proteins having an isoelectric point (p*I*) value of >7.5 (Nowak et al. 2011), and usually between ~8 and 10 (Roalson and McCubbin 2003).

An attempt to clone the full length of selected S-RNases using the partial sequences obtained through the initial 3′RACE cloning has been successful for two alleles. The full-length cloning of the S_*o2*_-RNase from *S. okadae* and S_*s2*_-RNase from *S. tuberosum* Group Stenotomum allowed the sequences of the 5′ regions to be determined. The full-length sequences revealed the two conserved catalytic histidine residues involved in the ribonuclease activity of S-RNases and located in the C2 and C3 regions (Fig. 4). Also, the eight conserved cysteine residues could be observed in S_*s2*_-RNase and only seven for S_*o2*_-RNase which lacks one such residue as described earlier. A variable number of potential N-glycosylation sites can be identified in S-RNase sequences (e.g. Oxley et al. 1998; Qi et al. 2001). However, the analysis of solanaceous S-RNase sequence has identified one single conserved potential N-glycosylation site found in the C2 conserved region (Ioerger et al. 1991). This was observed to be conserved in the full-length sequence of the two cloned putative S-RNases reported here. The presence of this conserved glycosylation site could possibly be responsible for modulating the S-RNase ribonuclease activity (Ioerger et al. 1991). However, studies have shown that, the removal of the glycan-side chains did not alter the enzymatic activity of the S-RNase gene in vitro (Broothaerts et al. 1991) or its function in self-incompatibility in transgenic *Petunia inflata* (Karunanandaa et al. 1994).

### Solanaceous S-RNases exhibit allelic diversity and intraspecific sequence polymorphism

An early report of the extreme level of polymorphism at the S-locus of a narrow endemic species, *Oenothera organensis*, which was estimated to contain ca. 500 individuals (Emerson 1939), generated interest in the understanding of the population genetics of gametophytic SI. The low level of amino acid sequence similarity observed in the partial sequences of the S-RNases reported here (32.9 %) is consistent with earlier observations made for solanaceous S-RNases (Ioerger et al. 1990; McCubbin and Kao 2000). Ioerger et al. (1990) analysed the S-locus of three species of the Solanaceae and observed a low level of amino acid sequence similarity within a species as low as 40 %. This is consistent with this study, where the amino acid sequence similarity within a species was as low as 32.9–44.2 %. Polymorphism at the *S*-locus and the high level of sequence divergence in solanaceous S-RNases could partly account for these observations. Solanaceous S-allele polymorphism has led to some unexpected observations where S-alleles of one species or genus were found to be more closely related to alleles from another species or genus; a case of trans-specific or trans-generic evolution of these S-alleles (Ioerger et al. 1990). It is proposed that the S-RNase alleles are exceptionally old and have been inherited from a common ancestor and passed down to multiple descendant taxa, i.e. the S-allele lineage origins predate their current species origin. More recent work has extended the initial observations in the Solanaceae to the Rosaceae family, specifically in the genus *Prunus* (Sutherland et al. 2008). Polymorphism at the S-locus in angiosperms is a result of diversifying selection, the age of S-alleles and the absence of recombination at the S-locus which acts to preserve and maintain allelic variations at the S-locus (Ioerger et al. 1991; Richman and Kohn 2000; Igic et al. 2003).

### Phylogenetic analysis of solanaceous S-RNases

The use of molecular phylogenetic analysis tools has enabled robust phylogenetic trees to be constructed leading to the identification of the most basal lineages of angiosperms. With the help of phylogenetic studies, S-genes have been characterised allowing conclusions to be drawn about the evolutionary history of their sequences (Allen and Hiscock 2008). For instance, the use of phylogenetic tools has enabled Igic and Kohn (2001) to make predictions that GSI is ancestral to ~75 % of eudicots and that RNase-based self-incompatibility of the GSI system was the ancestral state of self-incompatibility system existing in the majority of dicots.

All the identified S-RNases known to be involved in the GSI reaction in plants belong to the T2-type Class III gene subfamily (Igic and Kohn 2001). Other non-S-RNases referred to as S-like-RNases have also been identified in many plant species. These S-like-RNases share domain structure with S-RNases and appear in phylogenetic analyses to be related to S-RNases but have no role in the self-incompatibility reaction. In an initial attempt to show that the cloned *Solanum* putative S-RNases are genuine S-RNases and not S-like-RNases, a phylogenetic tree was constructed using an alignment of the cloned S-RNases and selected S-like-RNases from the Solanaceae (Fig. 5). The observation from the phylogenetic analysis that the non-S-RNases (S-like I and S-like II-RNases) fall outside the S-RNase clade is as anticipated. S-like-RNases are known to be unlinked to the S-locus (and hence are unlikely to be involved in GSI) and may be involved in pathogen defence mechanisms or induced in response to phosphate starvation (Kao and McCubbin 1996; Dodds et al. 1996; Hugot et al. 2002). The evolutionary relationship between S-RNases and S-like-RNases still remains uncertain, the S-like-RNases may be the ancestral genes involved in defence against pathogen attack in the style that were recruited with modifications to function in the self-incompatibility reaction (Kao and McCubbin 1996). Although rare examples of S-unlinked sequences, such as RNase X2 in *Petunia inflata*, are known to cluster in S-RNase clades (e.g. Lee et al. 1992), all the S-RNases cloned from this study appear to represent genuine S-alleles and are clearly distinct from and distantly related to S-like-RNases.

The use of phylogenetic tools has enabled us to compare the cloned S-RNases with other alleles from the Solanaceae to study the diversity of these S-RNases. A general observation is that the S-RNases do not form species-specific clades (Fig. 6) but rather that interspecific clades (showing interspecific similarities) are predominant. Similar results were obtained by Ioerger et al. (1990) when they analysed the S-locus of three species of the Solanaceae and observed a higher interspecies similarity rather than intraspecies similarity. They concluded that polymorphism at the S-locus predates the divergence of the species in the Solanaceae, and this polymorphism has been maintained to the present time. This extreme level of polymorphism in S-proteins indicates an unusual aspect of balancing selection which operates at the S-locus (Ioerger et al. 1990).

Furthermore, from the phylogenetic tree (Fig. 6), some trans-generic clades can be observed. For instance, S-RNases from *Solanum* (potato, tomato), *S. carolinense*, *Lycium*, *Petunia* and *Nicotiana* can be observed to form extensive trans-generic clades indicating extensive diversification of S-alleles in all these genera. In contrast, reduced or very limited trans-generic lineages could be observed for S-RNases from *Witheringia* and *Physalis*. This is consistent with previous studies that have revealed a clustering together of the *Physalis* alleles in just three clades of the extensive Solanaceae S-allele phylogenetic tree (Richman et al. 1996; Richman and Kohn 1999). Richman et al. (1996) proposed that the loss of trans-generic lineages in *Physalis crassifolia* was an outcome of the effect of severe population bottlenecks imposed on this genus. Similar explanations have been proposed to account for the reduced or very limited trans-generic lineages observed in the closely related genus *Witheringia* (Stone and Pierce 2005).

Following the identification of the first S-protein sequence from *Nicotiana* (Anderson et al. 1986), a large number of S-RNase sequences have been isolated from other species of the Solanaceae. However, unlike some other members of the Solanaceae, relatively few S-RNase gene sequences are available for potato. The relatively large number of putative S-RNases identified from the relatively small number of potato genotypes from this current study implies that there is a high level of S-RNase gene variability and diversity in potato. However, this S-gene variability has not been well exploited and characterised compared to other species in the Solanaceae. It is worth noting that although functional assays (e.g. transgenic) have not been performed for the cloned S-RNases, their sequence characterisation coupled with selected pollination tests in *Solanum okadae* indicates that they are likely to be genuine S-RNases and in the case of those tested by pollination clearly function in the SI reaction. The S-RNase genes reported here represent unique and useful additions to the limited available potato S-RNase gene sequence database. The identified alleles can be used for further studying the diversity and phylogenetic relationship of S-alleles, particularly in tuber-bearing *Solanum* (subsection *Petota*). These findings may also have application for the maintenance and application of potato germplasm for crop improvement.

## Electronic supplementary material

Below is the link to the electronic supplementary material.

**Supplementary Fig.1 RT-PCR cloning of pistil S-RNases in** ***Solanum okadae*** **OKA 9.** Colony PCR screening for OKA 9 pistil S-RNase using M13 universal primers. Lanes 1-13 represent individual transformed colonies and lanes labelled HP represent Hyperladder II (Bioline). Colonies which gave a PCR product of ~900 bp (*e.g.* colonies 3, 4, 6 & 12) have the expected insert size allowing for vector sequences. (DOCX 29 kb)

**Supplementary Fig.2 Colony PCR using M13 universal primers for OKA 1 (So2-RNase) 5’RACE.** Lanes1-11 represent transformed colonies and lanes labelled HP represent Hyperladder II (Bioline). Colonies which gave a PCR product of ~600 bp (*e.g.* colonies 3, 4 ,7, 8, 10 and 11) have the expected insert size. (DOCX 30 kb)

Supplementary material 3 (DOCX 45 kb)
