# Supplementary figures and images for: Allelic diversity of S-RNase alleles in diploid potato species
Source: Theor Appl Genet. 2016 Aug 6;129(10):1985–2001. doi: 10.1007/s00122-016-2754-7 (PMC5025496; doi:10.1007/s00122-016-2754-7)

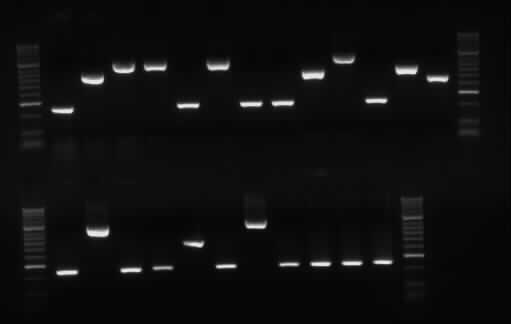


**HP 1 2 3 4 5 6 7 8 9 10 11 12 13 HP**

**~900 bp**

Supplement: Supplementary file 1 — Supplementary Fig.1 RT-PCR cloning of pistil S-RNases in Solanum okadae OKA 9. Colony PCR screening for OKA 9 pistil S-RNase using M13 universal primers. Lanes 1-13 represent individual transformed colonies and lanes labelled HP represent Hyperladder II (Bioline). Colonies which gave a PCR product of ~900 bp (e.g. colonies 3, 4, 6 & 12) have the expected insert size allowing for vector sequences. (DOCX 29 kb) [file 122_2016_2754_MOESM1_ESM.docx]

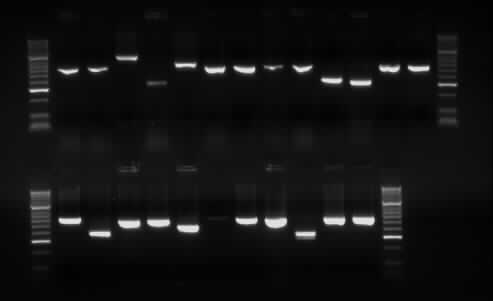


**HP 1 2 3 4 5 6 7 8 9 10 11 HP**

**~600 bp**

Supplement: Supplementary file 2 — Supplementary Fig.2 Colony PCR using M13 universal primers for OKA 1 (So2-RNase) 5’RACE. Lanes1-11 represent transformed colonies and lanes labelled HP represent Hyperladder II (Bioline). Colonies which gave a PCR product of ~600 bp (e.g. colonies 3, 4 ,7, 8, 10 and 11) have the expected insert size. (DOCX 30 kb) [file 122_2016_2754_MOESM2_ESM.docx]
