# Supplementary material for: Allelic diversity of S-RNase alleles in diploid potato species
Source: Theor Appl Genet. 2016 Aug 6;129(10):1985–2001. doi: 10.1007/s00122-016-2754-7 (PMC5025496; doi:10.1007/s00122-016-2754-7)
Supplement: Supplementary file 3 — Supplementary material 3 (DOCX 45 kb) [file 122_2016_2754_MOESM3_ESM.docx]

**Supplementary Tables**

**Supplementary Table S1 - Pollination data for *Solanum okadae***

| Female Parent | (X) | Male Parent | Berries | Seeds | Average Seed/Berry |
| --- | --- | --- | --- | --- | --- |
| OKA 1 | X | OKA 1 | 0 | 0 | 0 |
| OKA 1 | X | OKA 3 | 0 | 0 | 0 |
| OKA 1 | X | OKA 5 | 9 | 950 | 106 |
| OKA 1 | X | OKA 7 | 8 | 1000 | 125 |
| OKA 1 | X | OKA 9 | 12 | 1500 | 125 |
| OKA 3 | X | OKA 1 | 0 | 0 | 0 |
| OKA 3 | X | OKA 3 | 0 | 0 | 0 |
| OKA 3 | X | OKA 5 | 14 | 700 | 50 |
| OKA 3 | X | OKA 7 | 17 | 1200 | 71 |
| OKA 3 | X | OKA 9 | 14 | 1150 | 82 |
| OKA 5 | X | OKA 1 | 9 | 172 | 19 |
| OKA 5 | X | OKA 3 | 10 | 600 | 60 |
| OKA 5 | X | OKA 5 | 0 | 0 | 0 |
| OKA 5 | X | OKA 7 | 11 | 412 | 37 |
| OKA 5 | X | OKA 9 | 7 | 611 | 87 |
| OKA 7 | X | OKA 1 | 7 | 365 | 52 |
| OKA 7 | X | OKA 3 | 4 | 200 | 50 |
| OKA 7 | X | OKA 5 | 8 | 850 | 106 |
| OKA 7 | X | OKA 7 | 0 | 0 | 0 |
| OKA 7 | X | OKA 9 | 10 | 900 | 90 |
| OKA 9 | X | OKA 1 | 15 | 1700 | 113 |
| OKA 9 | X | OKA 3 | 20 | 2300 | 115 |
| OKA 9 | X | OKA 5 | 12 | 1200 | 100 |
| OKA 9 | X | OKA 7 | 17 | 1350 | 79 |
| OKA 9 | X | OKA 9 | 0 | 0 | 0 |

**Supplementary Table S2** - **Solanaceous S-like RNases used for the phylogenetic studies**

| S-like RNase | Class | Accession # | Species |
| --- | --- | --- | --- |
| NE | I | U13256.1 | *Nicotiana alata* |
| NGR3 | I | AB032257 | *Nicotiana glutinosa* |
| NK1 | I | AB034638 | *Nicotiana tabacum* |
| LE | I | X79337 | *Solanum lycopersicon* |
| RNS2 | II | AK324819.1 | *Solanum lycopersicon* |
| NGR2 | II | AB032256 | *Nicotiana glutinosa* |
| LER | II | CAL64053 | *Solanum lycopersicon* |

**Supplementary Table S3** - **Solanaceous S-RNases used for the phylogenetic studies**

| S-RNase | Accession # | Species |
| --- | --- | --- |
| S1 | DQ367853 | *Lycium parishii* |
| S2 | DQ367854 | *Lycium parishii* |
| S3 | DQ367855 | *Lycium parishii* |
| S4 | DQ367856 | *Lycium parishii* |
| S5 | DQ367857 | *Lycium parishii* |
| S5 | DQ367858 | *Lycium parishii* |
| S7 | DQ367859 | *Lycium parishii* |
| S8 | DQ367860 | *Lycium parishii* |
| S9 | DQ367861 | *Lycium parishii* |
| S10 | DQ367862 | *Lycium parishii* |
|  |  |  |
| S2 | X03803 | *Nicotiana alata* |
| S3 | U66427 | *Nicotiana alata* |
| S5 | GQ375151 | *Nicotiana alata* |
| S6 | U08861 | *Nicotiana alata* |
| Sc10 | U45959 | *Nicotiana alata* |
| S27 | GQ375153 | *Nicotiana alata* |
| S70 | GQ375150 | *Nicotiana alata* |
| S75 | GQ375152 | *Nicotiana alata* |
| S107 | GQ375154 | *Nicotiana alata* |
| S210 | GQ375155 | *Nicotiana alata* |
|  |  |  |
| S1 | M67990 | *Petunia inflata* |
| S2 | AY136628 | *Petunia inflata* |
| S3 | M67991 | *Petunia inflata* |
| S6 | AF301167 | *Petunia inflata* |
| S7 | AF301168 | *Petunia inflata* |
| S8 | AF301169 | *Petunia inflata* |
| S9 | AF301170 | *Petunia inflata* |
| S10 | AF301171 | *Petunia inflata* |
| S11 | AF301172 | *Petunia inflata* |
| S12 | AF301173 | *Petunia inflata* |
|  |  |  |
| S1 | AF281180 | *Physalis longefolia* |
| S2 | AF281181 | *Physalis longefolia* |
| S3 | AF281182 | *Physalis longefolia* |
| S4 | AF281183 | *Physalis longefolia* |
| S5 | AF374420 | *Physalis longefolia* |
| S6 | AF281184 | *Physalis longefolia* |
| S7 | AF281185 | *Physalis longefolia* |
| S8 | AF281186 | *Physalis longefolia* |
| S9 | AF281187 | *Physalis longefolia* |
| S10 | AF374421 | *Physalis longefolia* |

**Supplementary Table S3** (continuation)

| S-RNase | Accession # | Species |
| --- | --- | --- |
| A-SC | L40539 | *Solanum carolinense* |
| B-SC | L40540 | *Solanum carolinense* |
| C-SC | L40541 | *Solanum carolinense* |
| D-SC | L40542 | *Solanum carolinense* |
| E-SC | L40543 | *Solanum carolinense* |
| F-SC | L40544 | *Solanum carolinense* |
| G-SC | L40545 | *Solanum carolinense* |
| H-SC | L40546 | *Solanum carolinense* |
| J-SC | L40547 | *Solanum carolinense* |
| K-SC | L40548 | *Solanum carolinense* |
|  |  |  |
| S1 | EF680106 | *Solanum chilense* |
| S2 | EF680089 | *Solanum chilense* |
| S3 | EF680103 | *Solanum chilense* |
| S4 | EF680094 | *Solanum chilense* |
| S6 | EF680086 | *Solanum chilense* |
| S7 | EF680109 | *Solanum chilense* |
| S8 | EF680085 | *Solanum chilense* |
| S9 | EF680093 | *Solanum chilense* |
| S10 | EF680088 | *Solanum chilense* |
| S11 | EF680110 | *Solanum chilense* |
|  |  |  |
| S1 | AY454099 | *Witheringia solanacea* |
| S2 | AY454100 | *Witheringia solanacea* |
| S3 | AY454115 | *Witheringia solanacea* |
| S4 | AY454102 | *Witheringia solanacea* |
| S5 | AY454103 | *Witheringia solanacea* |
| S6 | AY454104 | *Witheringia solanacea* |
| S7 | AY454105 | *Witheringia solanacea* |
| S8 | AY454106 | *Witheringia solanacea* |
| S9 | AY454107 | *Witheringia solanacea* |
| S11 | AY454109 | *Witheringia solanacea* |

**Supplementary Table S4 – novel S-RNases reported in this study**

| S-RNase | Accession # | Species |
| --- | --- | --- |
| So1  So2  So3  So4  So5  Sp1  Sp2  Ss1  Ss2  Ss3  Ss4  Ss5  Ss6  Ss7  Ss8  Ss9  Ss10 | KX641173  KX641174  KX641175  KX641176  KX641177  KX641178  KX641179  KX641180  KX641181  KX641182  KX641183  KX641184  KX641185  KX641186  KX641187  KX641188  KX641189 | *Solanum okadae*  *Solanum okadae*  *Solanum okadae*  *Solanum okadae*  *Solanum okadae*  *S. tuberosum* Group Phureja  *S. tuberosum* Group Phureja  *S. tuberosum* Group Stenotomum  *S. tuberosum* Group Stenotomum  *S. tuberosum* Group Stenotomum  *S. tuberosum* Group Stenotomum  *S. tuberosum* Group Stenotomum  *S. tuberosum* Group Stenotomum  *S. tuberosum* Group Stenotomum  *S. tuberosum* Group Stenotomum  *S. tuberosum* Group Stenotomum  *S. tuberosum* Group Stenotomum |
